# Arginine and the metabolic regulation of nitric oxide synthesis in cancer

**DOI:** 10.1242/dmm.033332

**Published:** 2018-08-06

**Authors:** Rom Keshet, Ayelet Erez

**Affiliations:** Department of Biological Regulation, Weizmann Institute of Science, Rehovot 7610001, Israel

**Keywords:** Nitric oxide metabolism, Arginine, Cancer metabolism

## Abstract

Nitric oxide (NO) is a signaling molecule that plays important roles in diverse biological processes and thus its dysregulation is involved in the pathogenesis of various disorders. In cancer, NO has broad and sometimes dichotomous roles; it is involved in cancer initiation and progression, but also restricts cancer proliferation and invasion, and contributes to the anti-tumor immune response. The importance of NO in a range of cellular processes is exemplified by its tight spatial and dosage control at multiple levels, including via its transcriptional, post-translational and metabolic regulation. In this Review, we focus on the regulation of NO via the synthesis and availability of its precursor, arginine, and discuss the implications of this metabolic regulation for cancer biology and therapy. Despite the established contribution of NO to cancer pathogenesis, the implementation of NO-related cancer therapeutics remains limited, likely due to the challenge of targeting and inducing its protective functions in a cell- and dosage-specific manner. A better understanding of how arginine regulates the production of NO in cancer might thus support the development of anti-cancer drugs that target this key metabolic pathway, and other metabolic pathways involved in NO production.

## Abstract (precis)

**Summary:** In this Review, the authors focus on the regulation of NO levels by the synthesis and availability of arginine, and on the implications of this metabolic regulation to cancer biology and therapy.

## Introduction

Nitric oxide (NO) is a short-lived, gaseous signaling molecule that is produced endogenously by a family of enzymes called the nitric oxide synthases (NOS), which catalyze the synthesis of NO from the amino acid arginine (Bredt, 1999). NO regulates various signaling pathways in many different tissues and has diverse physiological roles. The most well-known and established functions of NO relate to its roles in the immune, cardiovascular and neuronal systems. Indeed, NO is produced by different immune cells, mainly macrophages, and is a key regulator of immunity and inflammation (Predonzani et al., 2015). It is required for the activation and migration of macrophages (Connelly et al., 2003; Maa et al., 2008) and, in infectious conditions, NO released by immune cells has cytotoxic antimicrobial activities (Woodmansee and Imlay, 2003). Conversely, NO can serve as an immunosuppressive agent that limits T-cell proliferation and activity by promoting apoptosis and by inhibiting cytokine and chemokine production (Bogdan, 2015). In the cardiovascular system, endothelium-derived NO is a powerful vasodilator and has a central role in setting vascular tone and blood pressure (Zhao et al., 2015). Moreover, NO is known to participate in vascular endothelial growth factor (VEGF)-induced vascular permeability and angiogenesis (the formation of new blood vessels) (Fraisl, 2013). Finally, in the nervous system, neuronal-derived NO is known to regulate neural development (Kong et al., 2014) and to influence various brain functions, such as cognition and response to stress (Philippu, 2016). In the peripheral nervous system, NO regulates the function of nerves that regulate smooth-muscle tone and motility in the gastrointestinal tract (Toda and Herman, 2005). Thus, NO regulates neuronal and blood-vessel functions in most tissues and, as such, is essential for the preservation of physiological homeostasis. Indeed, unregulated NO production is implicated in multiple pathophysiological conditions, including cancer (Burke et al., 2013).

In this Review, we summarize what is known about NO metabolism in carcinogenesis, focusing on the importance of arginine synthesis and its availability for NO production. Additionally, we discuss the role of the arginine-NO axis in cancer biology and its potential implications in developing NO-related cancer therapeutics. An improved understanding of this metabolic pathway might enable current treatments to be optimized and would support the development of other novel anti-cancer drugs that target tumors in which NO plays a central role in disease initiation and progression.

## NO synthesis and metabolism

In mammals, three distinct genes encode the three NOS isoforms: neuronal NOS (nNOS; encoded by *NOS1*), inducible NOS (iNOS; encoded by *NOS2*) and endothelial NOS (eNOS; encoded by *NOS3*). *NOS1* and *NOS3* are constitutively expressed mainly in neurons and in endothelial cells, respectively, whereas *NOS2* is mainly expressed in immune cells (Mungrue et al., 2003). The binding of calcium (Ca^2^^+^) and calmodulin to nNOS and eNOS transiently activates them to produce nanomolar concentrations of NO, whereas iNOS expression is induced by inflammatory cytokines or by bacterial products, such as lipopolysaccharide (LPS), to produce micromolar concentrations of NO (Lind et al., 2017). Interestingly, in addition to cytosolic NOS, there is a mitochondrial variant of NOS that contributes to the regulation of NO-related mitochondrial activities (Ghafourifar and Richter, 1997). All NOS isoforms use arginine as a substrate and require oxygen, NADPH and the cofactor tetrahydrobiopterin (BH4) to generate NO and citrulline (see Box 1 for a glossary of terms) (Mungrue et al., 2003). In this reaction, electrons donated by NADPH at the carboxy-terminal reductase domain of NOS are passed to the heme catalytic center of the oxidase domain, where activation of molecular oxygen is ‘coupled’ to NO synthesis by two successive mono-oxygenations of arginine. NO can also be generated from the inorganic anions, nitrate (NO_3_^−^) and nitrite (NO_2_^−^), particularly in hypoxic states (Lundberg et al., 2008). This pathway has been discussed extensively in previous reviews (Lundberg et al., 2008), and hence will not be discussed further here.

**Box 1. Glossary**

**Anchorage independence:** the capacity of cancer cells to divide and function despite the absence of a stable surface to anchor to.

**Epithelial-to-mesenchymal transition (EMT):** a process by which epithelial cells lose their cell polarity and cell-cell adhesion, and gain migratory and invasive properties to become mesenchymal-like cells.

**NO donor:** a molecule that induces nitric oxide (NO) under physiological conditions.

**S-nitrosylation:** a protein post-translational modification in which NO is covalently attached to cysteine residues to form S-nitrosocysteine.

**Tetrahydrobiopterin (BH4):** a naturally occurring cofactor that is required by nitric oxide synthase (NOS) enzymes to produce NO, as well as by other enzymes involved in amino acid degradation and in neurotransmitter synthesis.

Cellular NO levels are tightly regulated at several different levels and by multiple factors. NO can be regulated at the level of NOS transcription or via the post-translational modification of NOS, as well as via the cellular expression of the different NOS isoforms, and through the availability of NOS substrates, such as arginine and BH4 (Mungrue et al., 2003). It can also be regulated by the amount of NO produced by the different NOS, as well as by the short half-life of NO, which is estimated to be in the range of 0.1–2 s and allows the rapid termination of NO signaling cascades once the initial stimulus is turned off (Thomas et al., 2001).

The biological effects of NO are exerted through either cyclic guanosine monophosphate (cGMP) or via post-translational modification (PTM) by S-nitrosylation (Box 1) (Seth et al., 2018). Canonical NO signaling involves soluble guanylate cyclase (sGC), which is the only known receptor for NO. This enzyme is a heterodimer composed of two subunits, one of which contains a heme group to which NO binds and activates the enzyme (Martin et al., 2000). This reaction leads to the production of cGMP and brings about the activation of cGMP-dependent kinases, which transduce multiple signaling events through protein phosphorylation (Murad, 2006). Non-canonical NO signaling is achieved mainly by S-nitrosylation. In this reaction, NO covalently binds to alkyl sulfur atoms on proteins and organic compounds, without the assistance of enzymes, to form S-nitrosothiols. This reaction requires higher concentrations of NO and tends to proceed with slower kinetics than cGMP-mediated actions. S-nitrosylation impacts protein function, stability and localization by modulating the cysteine-containing active sites of enzymes and by regulating protein-protein interactions through altering the affinity of cysteine-containing binding niches (Hess et al., 2005; Gould et al., 2013; Doulias et al., 2013).

NO can be further metabolized to form reactive nitrogen species, such as peroxynitrite (OONO^−^), which have distinctive physiological and pathological roles of their own (Adams et al., 2015). Peroxynitrite forms when NO reacts with superoxide; it is released from immune cells to assist with pathogen killing via the oxidization of protein residues. However, the overproduction or dysregulation of peroxynitrite levels can lead to a chronic inflammatory response (Adams et al., 2015). In addition, NOS generates superoxide and hydrogen peroxide when the concentrations of arginine and BH4 are low (Porasuphatana et al., 2003). For example, when vascular BH4 levels are limited, electron flow to molecular oxygen becomes ‘uncoupled’ from arginine oxidation, resulting in the generation of superoxide anion and of other reactive oxygen species (ROS), rather than in the generation of NO. Both superoxide anions and ROS contribute to the pathogenesis of vascular disease (Chuaiphichai et al., 2017) and to cancer progression, as will be further discussed below (Rabender et al., 2015).

Thus, NO-dependent signaling pathways are highly complex, and the proper regulation of NO production is vital for executing the functions of these pathways. Central to this regulation is the production and availability of the NO precursor arginine.

## NO regulation by arginine metabolism

**Fig. 1.**
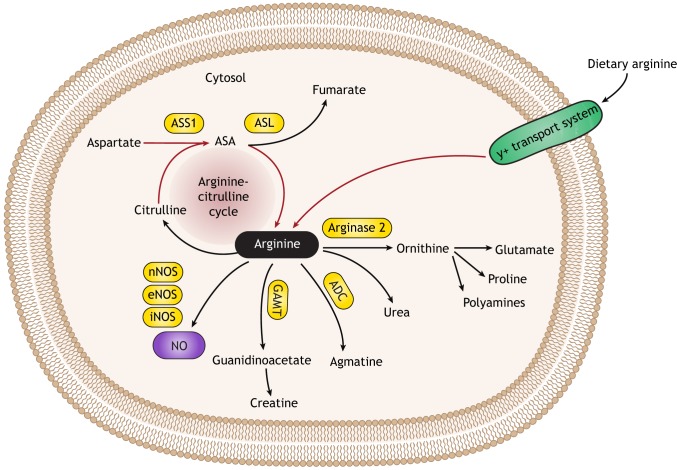
**A schematic illustration of arginine metabolism outside of the liver.** Arginine from dietary intake can enter a cell via the y+ transport system or it can be synthesized endogenously by the arginine-citrulline cycle (red arrows). In contrast to the single enzyme that synthesizes arginine (ASL), many enzymes utilize arginine as their substrate (black arrows), to synthesize a range of compounds, including ornithine, agmatine, guanidinoacetate and NO, in accordance with cellular needs. NO is synthesized from arginine by either one or by all three NOS isoforms (eNOS, iNOS or nNOS), depending on cellular context. ADC, arginine decarboxylase; ASA, argininosuccinic acid; ASL, argininosuccinate lyase; ASS, argininosuccinate synthase 1; eNOS, endothelial nitric oxide synthase; GAMT, guanidinoacetate methyltransferase; iNOS, inducible nitric oxide synthase; NO, nitric oxide; nNOS, neuronal nitric oxide synthase.

Although the physiological intracellular concentrations of arginine far exceed those required for eNOS to synthesize NO, the acute provision of exogenous arginine increases NO production (Dioguardi, 2011). This phenomenon is known as the ‘arginine paradox’. The arginine pool derives from several sources, including dietary intake, body protein breakdown and endogenous *de novo* synthesis (Fig. 1). Endogenous, systemic arginine production occurs through the intestinal-renal axis, in which citrulline produced by the intestine is converted into arginine by the kidneys. Here, citrulline availability is the limiting factor for the amount of arginine synthesized (Wu et al., 2009). To supply tissues with their required arginine needs, circulating arginine traverses cell membranes via the Na^+^-independent, cationic amino acid transport system y^+^, which regulates the availability of arginine for arginine-dependent synthetic pathways (Arancibia-Garavilla et al., 2003). Of note, arginine is also produced as an intermediate in the liver urea cycle, but here it participates in the detoxification of excess nitrogen; as such, it is not secreted into the plasma and does not contribute to the body's pool of arginine (Watford, 1991; Wu et al., 2009).

Arginine is a semi-essential amino acid, meaning that, under physiological conditions, its endogenous synthesis is sufficient to meet the body requirements, and no additional supplementation is required from diet. In certain physiological and pathological states, such as during infancy, growth, pregnancy and illness, such as infections and cancer, arginine is synthesized endogenously in multiple tissues by the arginine-citrulline cycle. This is because the amount of circulating arginine, as derived from dietary intake and kidney production, cannot meet cellular requirements for arginine during these states (Fig. 1). The arginine-citrulline cycle operates in most mammalian cell types where arginine is generated to meet cellular needs for its downstream metabolites (Husson et al., 2003). Indeed, arginine is a major metabolic nexus for the synthesis of multiple metabolites, among which are NO, polyamines, proline and creatine, all of which are essential for cell survival and proliferation (Rhee et al., 2007; Liang et al., 2013; Sestili et al., 2016). Besides NOS, the other two enzymes that function in the arginine-citrulline cycle are argininosuccinate synthase 1 (ASS1) and argininosuccinate lyase (ASL), both of which also function in renal arginine production and in the liver as part of the urea cycle. ASS1 is a cytosolic enzyme that catalyzes the formation of argininosuccinate from citrulline and aspartate, with ATP being broken down into AMP and pyrophosphate during the reaction. Subsequently, ASL promotes the cleavage of argininosuccinate to arginine and fumarate. Arginine can then be recycled back to citrulline by NOS or be utilized by other enzymes for the synthesis of either ornithine, agmatine or guanidinoacetate. In contrast to the four cellular enzymes that use arginine as a substrate, only ASL can generate endogenous arginine in mammalian cells (Fig. 1). Thus, another important factor that regulates the intracellular availability of arginine for NOS is the activity of other competing enzymes that also use arginine as a substrate. One of the main competitors is another urea cycle enzyme, arginase, which converts arginine into ornithine and urea, thereby limiting the availability of arginine for NOS (Caldwell et al., 2015). There are two isozymes of this enzyme, arginase-1 (ARG1), which functions in the urea cycle and is located primarily in hepatocytes, and arginase-2 (ARG2), which is ubiquitously expressed outside of the liver, where it competes with NOS for arginine (Caldwell et al., 2015). Importantly, in response to an inflammatory stimulus like LPS, activated (M1) macrophages express iNOS, whereas, during inflammation resolution, macrophages switch to express ARG1, which sequesters arginine from iNOS. This switch in gene expression leads to the increased production of ornithine and its downstream metabolites, polyamines and proline, with a subsequent decrease in NO production (Weisser et al., 2013).

Thus, various factors determine the availability of intracellular arginine in the specific cellular compartment for the synthesis of NO, including dietary intake of arginine, the expression of its transporters, its level of synthesis by ASS1 and ASL, and its competing use as a substrate by other enzymes. These multiple variables impact NO levels during homeostasis, as well as in different disease states, such as cancer (Wu et al., 2009).

## Dichotomous roles of NO in cancer

**Fig. 2.**
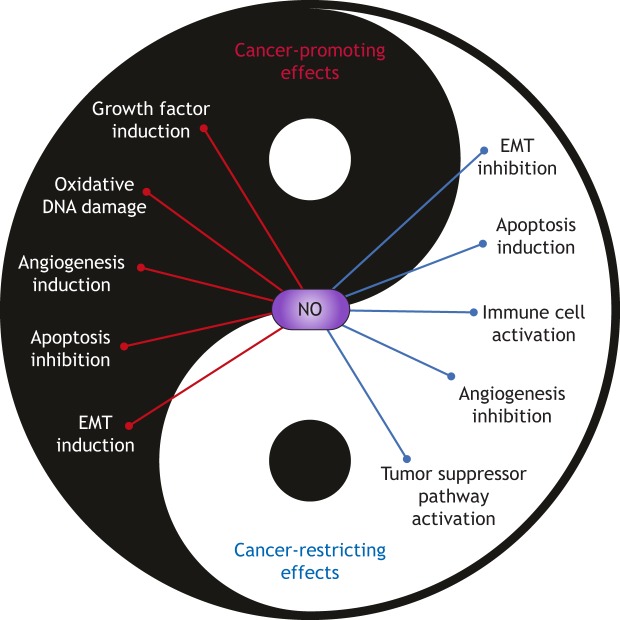
**Dichotomous roles for NO in cancer.** The dichotomous roles of NO in cancer are depicted in a Yin and Yang model, to illustrate its cancer-promoting and cancer-restricting effects. NO can either inhibit or enhance cancer progression, depending on the biological context, its concentration and on the duration of NO production. EMT, epithelial-to-mesenchymal transition; NO, nitric oxide.

NO has been linked to the pathogenesis of different tumor types, functioning as either an enhancer or inhibitor of cancer development (Fig. 2). The contribution of NO to cancer progression includes the activation of mitogenic pathways. Treating cultured human breast cancer cells with an NO donor (Box 1) resulted in the activation of the epidermal growth factor receptor (EGFR) and of the extracellular signal-regulated kinase (ERK) pathways through EGFR S-nitrosylation, with a subsequent increase in the migration and invasive potential of these cells (Garrido et al., 2017). Similarly, the mTOR mitogenic pathway has been shown to be activated by NO via the S-nitrosylation of key proteins to promote the proliferation of human melanoma cells, both *in vitro* and in an animal xenograft models (Lopez-Rivera et al., 2014). Another important oncogenic pathway in cancer promoted by NO to induce proliferation and migration is the Wnt/β-catenin pathway, as suggested by the activation of Wnt target genes following the overexpression of iNOS in cultured human colon and breast cancer cells (Du et al., 2013). Interestingly, NO can also support tumor-forming cancer stem cells (CSCs), as NO produced in cultured human colon CSCs was found to drive stemness-related signaling pathways, central to colon tumor initiation and progression (Puglisi et al., 2015). In contrast to the role that NO plays in supporting oncogenic pathways in cancer, NO also exhibits an anti-proliferative role by suppressing oncogenic pathways or by activating tumor-suppressing ones. Indeed, NO has been shown to negatively regulate the proliferation of human neuroblastoma cell lines by decreasing the expression of the oncogene c-Myc in a cGMP-dependent manner (Ciani et al., 2004). Moreover, NO can inhibit the proliferation of a human neuroblastoma cancer cell line *in vitro* by upregulating tumor suppressor pathways, including the BRCA1/Chk1/p53 pathway, leading to cell cycle arrest in response to DNA damage via activation of cell cycle checkpoints (Van de Wouwer et al., 2012). Of note, the dual roles of NO in cancer are also dose-dependent; while exogenous NO stimulated cell proliferation in pheochromocytoma PC12 cells at low concentrations, it inhibited proliferation at higher concentrations (Bal-Price et al., 2006). Likewise, whereas low levels of NO can inhibit apoptosis and promote cancer, high levels of NO can contribute to cancer cell apoptosis (Villalobo, 2006).

NO has also been implicated in the epigenetic modification of gene expression. Several studies have described NO-driven epigenetic modifications that control normal biological development and mediate tumorigenesis (Vasudevan et al., 2016). In prostate carcinogenesis, the silencing of glutathione transferase P1-1 (*GSTP1*) is a common early event that is frequently caused by promoter hypermethylation and correlates with decreased survival. In human prostate cancer cells, eNOS participates in *GSTP1* repression by being recruited to the gene promoter with a consequential remodeling of the local chromatin (Re et al., 2011). Importantly, pharmacological inhibition of eNOS relieved the repression of *GSTP1*, and treatment with an NO donor silenced this gene, suggesting that eNOS regulates *GSTP1* transcription through NO production. In oral squamous cell carcinoma patients, it is common to find histone hyperacetylation that promotes tumor progression. Interestingly, it has been found that NO mediates histone hyperacetylation and that p300 histone acetylase activity is dependent on endogenously generated NO (Arif et al., 2010). Moreover, NO can affect histone PTMs at a global level, as treatment with NO donors resulted in the differential expression of over 6500 genes in breast cancer cells, in which the pattern of PTMs correlated with an oncogenic signature (Vasudevan et al., 2015). Conversely, NO has been found to inhibit lysine demethylase 3A (KDM3A), a histone demethylase that is known to positively regulate cancer cell invasion, chemoresistance and metastasis in breast and ovarian cancer cells (Hickok et al., 2013).

The tumor microenvironment also influences the contribution of NO to tumor fate. Indeed, cancer cells have developed diverse ways to intervene in the production and/or metabolism of NO in tumors and their surrounding tissue to gain an advantage. As with its dichotomous roles in cancer cell proliferation and apoptosis, NO can influence both cancer progression and its restriction. *NOS1* upregulation was found to support the growth and activity of cultured cancer-associated fibroblasts, which are known to stimulate tumor progression (Augsten et al., 2014). Additionally, NO was demonstrated to directly promote cancer progression and invasion by influencing the stromal components of a tumor, by inducing epithelial-to-mesenchymal transition (EMT; Box 1), as well as by affecting tumor vessel formation. In human squamous cell carcinoma and in lung cancer cells lines, EMT and stem cell features are reportedly activated by moderate levels of NO produced by NOS in response to growth factors and inflammatory mediators (Terzuoli et al., 2017). On the other hand, it seems that, in the same cells, higher than normal concentrations of NO inhibit EMT. In human metastatic prostate cancer cell lines treated with a high concentration of the NO donor DETA-NONOate, EMT and the invasive phenotype of these cells is reversed via the inhibition of the EMT effector and transcription factor Snail (Baritaki et al., 2010). It was demonstrated in this study that both Snail mRNA levels and its DNA-binding capacity were inhibited by NO, in a yet-undefined mechanism. Moreover, NO was found to inhibit the mitochondrial function of Complex I and IV of the electron transport chain (Dai et al., 2013), and to perturb the integrity of the mitochondrial network by S-nitrosylation of dynamine-1-like protein (DRP-1), which is known to regulate mitochondrial fission (Cho et al., 2009). It is thus tempting to speculate that the proliferation and EMT elicited by low doses of NO in cancer are associated with the inhibition of mitochondrial activity (Boland et al., 2013).

Cancer progression also depends on angiogenesis, which is needed to support the growing tumor with oxygen and nutrients, and to remove waste products. NO can promote angiogenesis by supporting endothelial differentiation, by inhibiting antiangiogenic factors, by dilating tumor blood vessels, and by recruiting bone-marrow-derived and perivascular cells (Fukumura et al., 2006). In a murine melanoma model lacking eNOS expression, a perturbed recruitment of mural cells to newly formed vessels, and abnormal vessel branching and stabilization were demonstrated (Kashiwagi et al., 2005). Moreover, nNOS was required for the formation of abnormal tumor blood vessels in mice harboring human glioma xenografts (Kashiwagi et al., 2008). Conversely, several NO-related metabolites, such as isosorbide mononitrate and dinitrate, have been found to suppress VEGF protein levels in cultured human colon cancer cells, and to inhibit angiogenesis *in vivo* in xenografts of murine lung tumors (Pathi et al., 2011; Pipili-Synetos et al., 1995).

In recent years, researchers have focused on the role of the immune system in cancer development. Here too, NO produced by immune cells has dual regulatory functions in tumor progression. NO and reactive nitrogen species that originate from immune cells, such as macrophages and neutrophils, can have pro-tumorigenic effects on neighboring epithelial cells, for example, by inducing DNA damage that can initiate inflammation-associated neoplastic transformation (Wang et al., 2017). NO produced by tumor-infiltrating myeloid cells was found to be important for activation of adoptively transferred cytotoxic T cells (Marigo et al., 2016). It was also shown that NO production by colon cells is required in pathogen-induced colon inflammation and immune cell infiltration, eventually leading to dysplasia and colon cancer development (Erdman et al., 2009). In parallel, NO can activate macrophages and cytotoxic T cells, and augment the immune response against tumor cells (MacMicking et al., 1997; Marigo et al., 2016). Indeed, the cytotoxic action of the cytokine interferon gamma (IFN-γ), or the activity of LPS-activated primary mouse macrophages against different cancer cell lines, were impaired in macrophages from *NOS2* knock-out mice (MacMicking et al., 1997). Moreover, animal studies have demonstrated that, in tumor vessels of melanoma xenografts, macrophage-derived NO induced the expression of the adhesion molecule VCAM-1, which is important for T-cell extravasation. Additionally, only co-transfer of CD8^+^ T cells with wild-type macrophages, but not with *Nos2*^−/−^ macrophages, yielded T-cell homing to the tumor, and consequently led to tumor rejection (Sektioglu et al., 2016).

These studies collectively suggest that cellular levels of NO, and the associated cross-talk between cancer cells and their environment, are important for tumor initiation and progression. As a consequence, efforts have been made in recent years to improve NO detection and analysis (Box 2).

**Box 2. Detecting NO in cancer**

Cellular concentrations of nitric oxide (NO) are hard to quantify due to its very short half-life. In biological tissue samples, NO levels can be measured indirectly by quantifying nitrite and nitrate using high-performance liquid chromatography (HPLC) (Jiang et al., 2012). The reagent 4-amino-5-methylamino-2′,7′-difluorofluorescein (DAF-FM) is used for intracellular measurements because it becomes fluorescent when it reacts with NO and can be detected by any fluorescein-detecting instrument (Namin et al., 2013). Undoubtedly, the ideal method for measuring NO levels is via a flux analysis, performed *in vivo* by using labeled arginine and then tracing the downstream products, such as citrulline and NO-derived urinary nitrate, by using gas or liquid chromatography-mass spectrometry (Magné et al., 2009).

For a more global assessment of NO involvement in cancer, the expression of individual genes in the arginine-NO pathway can be assessed across different types of cancer in large datasets, such as in The Cancer Genome Atlas (TCGA). Expression levels can then be correlated with patient survival and therapeutic response (Ekmekcioglu et al., 2016).

Although these methods have all contributed to our understanding of NO biology, NO production and its downstream effects often involve complex signaling pathways and thus it remains challenging to dissect the exact cellular contribution of NO to disease pathogenesis and to investigate its therapeutic relevance.

## Metabolic regulation of NO synthesis in cancer

Cancer cells regulate the availability of intracellular arginine for NO synthesis in multiple ways. Cultured human colon cancer cells and murine breast cancer cells stimulated by inflammatory mediators, such as LPS and IFN-γ, increase the availability of arginine for NO production by enhancing transmembrane arginine import (Cendan et al., 1996a,b). Omental adipose stromal cells (O-ASCs) are mesenchymal stem cells contained in the omentum tissue that are known to promote endometrial and ovarian tumor proliferation. Interestingly, O-ASCs can support human endometrial or ovarian cancer cells in co-culture by supplying them with arginine for NO production, a finding that may reflect their role in tumor progression (Salimian Rizi et al., 2015). Another way to upregulate arginine levels in tumor cells is to increase its endogenous synthesis. In samples from colon and breast cancer patients, the overexpression of *ASL*, which encodes the enzyme that synthesizes arginine, is associated with poor survival (Huang et al., 2015, 2017a). Additionally, tumor samples from hepatocellular carcinoma patients show increased *ASL* expression and, in human colon, breast and hepatocellular carcinoma cells, silencing of *ASL* expression by short-hairpin RNA, or the reduction of NO production by a NOS inhibitor, inhibited cancer cells' proliferation and anchorage-independence (Box 1) (Huang et al., 2013, 2015, 2017a). These studies imply that ASL exerts its tumorigenic effects at least in part through NO, but they do not rule out the possibility that increased arginine levels might promote cancer by supporting the synthesis of other arginine-derived molecules, such as polyamines.

Another urea cycle enzyme that functions in the arginine-citrulline cycle, ASS1, is also overexpressed in various human cancers, including in lung, colon, gastric and ovarian cancer (Delage et al., 2010). The upregulated expression of *ASS1* has been found to support the proliferation of human colon cancer cells *in vitro*, as well as the migration and metastatic potential of human gastric cell lines, both *in vitro* and in mice with tumor xenografts (Bateman et al., 2017; Shan et al., 2015). Yet, the cancer-promoting mechanisms fostered by *ASS1* overexpression, and their clinical implications, remain unclear. It is possible that high levels of ASS1 support tumor proliferation and aggressiveness by increasing the supply of arginine for NO production. In support of this, *ASS1* overexpression in rat vascular smooth muscle cells potentiated the LPS- and IFN-γ-stimulated production of NO (Xie and Gross, 1997). Moreover, human breast cancer cells treated with the pro-inflammatory cytokine interleukin 17 (IL-17) increased their proliferation, and this response was reported to depend on the enhanced availability of arginine for NO production. This increased arginine flux was associated with the upregulated expression of both *ASS1* and *NOS3*, and with the downregulated expression of arginase (Amara et al., 2017).

*ASS1* expression can also be downregulated in some cancers in association with the methylation of its promoter (Wu et al., 2013). Reduced *ASS1* expression has been associated with higher recurrence, shorter disease-free survival and with shorter overall survival in patients with pancreatic cancer (Liu et al., 2017). In osteosarcoma patients, lower *ASS1* expression levels in tumor samples were associated with resistance to doxorubicin treatment (Kim et al., 2016) and with the development of pulmonary metastases (Kobayashi et al., 2010). Interestingly, *ASS1* and *ASL* expression was silenced by gene promoter methylation in primary cultures of human glioblastoma multiforme cells, suggesting that these genes are not mutually exclusive and, hence, silencing of each one of these genes may modulate a separate metabolic pathway (Syed et al., 2013). Indeed, independent of arginine importance, cancer cells become more proliferative when *ASS1* is silenced because of the increased cytosolic availability of its substrate, aspartate, for pyrimidine synthesis by CAD (carbamoyl-phosphate synthase 2, aspartate transcarbamylase and dihydroorotase) (Rabinovich et al., 2015; Moreno-Morcillo et al., 2017). Nevertheless, it is also possible that ASS1 downregulation promotes cancer by decreasing the availability of arginine for NO synthesis. Importantly, the silencing of *ASS1* or *ASL* in tumors results in arginine auxotrophy – an intrinsic dependence of the cells on exogenous arginine due to their inability to synthesize it. In these circumstances, arginine becomes an essential amino acid, generating a vulnerability that can be used to treat cancer using arginine-depriving agents (Syed et al., 2013).

Cancer cells can also increase NO production via the upregulation of NOS. Indeed, it has been demonstrated that, following the exposure of a human osteosarcoma cell line to LPS- or IFN-γ-induced iNOS, NO levels subsequently increased (Tachibana et al., 2000). Moreover, hypoxia and inflammatory cytokines can induce iNOS expression in cultured human breast cancer cells with a subsequent elevation in poor-survival biomarkers such as S100 calcium-binding protein A8, IL-6, IL-8 and tissue inhibitor of matrix metalloproteinase-1 (Heinecke et al., 2014). In addition, iNOS mRNA and protein levels were found to be elevated in tumor samples from patients with nasopharyngeal carcinoma (Segawa et al., 2008). Another interesting cancer-associated mechanism involving NOS is the reduction in the availability of BH4, which has been reported in human breast, colorectal, epidermoid, and head and neck tumors, as compared to normal human tissues (Rabender et al., 2015). As a consequence, rather than NO, NOS activity generates more peroxynitrite, which has anti-apoptotic signaling properties (Delgado-Esteban et al., 2007). Accordingly, treating human breast cancer cells with a BH4 precursor inhibited their growth, both in culture and in tumor xenografts *in vivo* (Rabender et al., 2015)*.* In addition to NO produced by cytosolic NOS, NO produced by the mitochondrial NOS has also been found to be relevant to cancer. Mitochondrial NO synthesis requires, at least in part, the transport of arginine to the mitochondria through the solute carrier family 25, member 29 (SLC25A29) transporter (Porcelli et al., 2014). The relevance of this diversion of arginine to mitochondrial NO synthesis is illustrated by the deleterious consequences of SLC25A29 deletion in cancer cells, which was found to impair NO production and to reduce tumor growth (Zhang et al., 2018). However, the regulation and molecular consequences of the cytoplasmic versus mitochondrial production of NO require further investigation.

The enzyme ARG2 competes with NOS for arginine as a substrate; accordingly, as mentioned above, the downregulation of ARG2 might increase the availability of arginine for NO production (Amara et al., 2017). In contrast, human breast tumor tissues express high levels of the *ARG2* gene, and its inhibition in breast cancer cell xenografts by the 3-hydroxy-3-methyl-glutaryl coenzyme A (HMG CoA) reductase inhibitor rosuvastatin led to the inhibition of tumor proliferation (Erbas et al., 2015; Singh et al., 2013). This therapeutic effect was suggested to be due to a reduction in the use of arginine as a precursor for polyamines, with a concomitant increase in its usage as a precursor for NO (Cervelli et al., 2014).

## NO-related drugs for cancer therapy

**Fig. 3.**
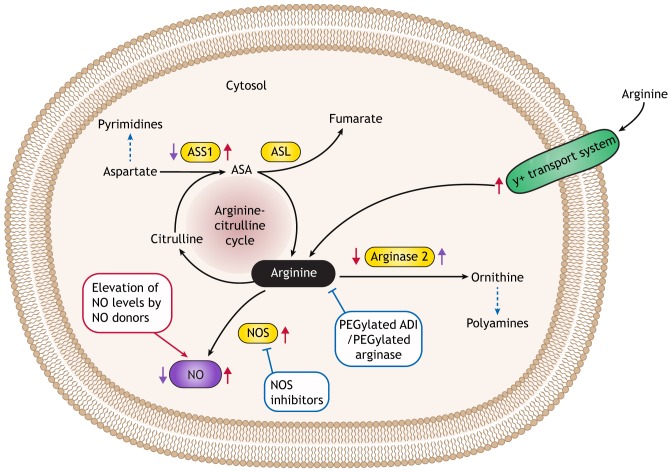
**NO-metabolism-related anti-cancer strategies.** A schematic illustration of NO metabolic pathways in a cancer cell. Red arrows denote the cancer-related up- or down-regulation of proteins that are involved in NO metabolism, leading to a net increase in NO production. Tumor cells can enhance NO production by: upregulating NOS levels; increasing arginine transport; increasing the levels of ASS1 and ASL to enhance arginine availability for NO synthesis; or by decreasing arginine metabolism by inhibiting arginase. Purple arrows denote the cancer-related up- or down-regulation of proteins involved in NO metabolism, leading to a net decrease in NO production. In addition to restricting NO levels, ASS1 inhibition and ARG2 upregulation might also metabolically support cancer by increasing the production of pyrimidines and polyamines, respectively (dashed blue arrows). Anti-cancer NO-related strategies that increase NO levels are denoted in a red box and those that downregulate NO levels are depicted in blue boxes. NO-related anti-cancer strategies include increasing levels of NO with NO donors, or decreasing NO levels via NOS inhibition or with PEGylated arginine-degrading enzymes. ADI, arginine deiminase; ASA, argininosuccinic acid; ASL, argininosuccinate lyase; ASS1, argininosuccinate synthase 1; NO, nitric oxide; NOS, nitric oxide synthase.

Because high NO levels have been shown to play a tumorigenic role in various types of cancer, one rational approach to treating such cancers is to develop drugs that decrease NO levels (Fig. 3). Several drugs that inhibit NOS enzymatic activity exist (Paige and Jaffrey, 2007). However, clinical trial results were complex. For example, NOS inhibition using drugs such as Sanggenon C or L-NG-nitroarginine methyl ester (L-NAME) restricted tumor growth in mouse xenograft cancer models (Chen et al., 2017; Pershing et al., 2016; Ridnour et al., 2015). Yet, a Phase 1 clinical trial of the iNOS inhibitor ASP9853, used in combination with the chemotherapeutic drug docetaxel to treat patients with resistant solid tumors, was prematurely terminated because of neutropenia-associated toxicities (Luke et al., 2016). Interestingly, the endogenous compound asymmetric dimethylarginine (ADMA), which inhibits NOS by competing with arginine, has recently been shown to be degraded by the enzyme dimethylarginine dimethylaminohydrolase-1 (DDAH1). DDAH1 is frequently upregulated in prostate cancer, where it promotes tumor growth and angiogenesis, suggesting that anti-cancer drugs that induce ADMA or that inhibit DDAH1 could potentially be useful in treating tumors that are influenced by the pro-tumorigenic properties of NO (Reddy et al., 2018).

As discussed above, arginine-depleting agents are being tested as treatment for tumors that are auxotrophic for arginine. The enzyme arginine deiminase (ADI), which allows many microorganisms to utilize arginine as a major energy source, was recently included in clinical trials as an anti-cancer drug to treat arginine-auxotrophic tumors, with positive effects reported on reducing disease progression in hepatocellular carcinoma, advanced pancreatic adenocarcinoma and acute myeloid leukemia patients (Izzo et al., 2004; Lowery et al., 2017; Tsai et al., 2017). Since NO is an important product of arginine in cancer cells, it is plausible that arginine depletion might contribute to tumor inhibition by reducing the cellular levels of NO. In Phase 1 and 2 clinical trials, metastatic melanoma patients responded to ADI treatment and showed reduced plasma NO levels; however, no causative effect was proven between plasma NO levels and the clinical response to treatment (Ascierto et al., 2005). Another arginine-depleting agent, a PEGylated derivative of recombinant human ARG1, was found to inhibit cancer progression when tested in a Phase 1 clinical trial for the treatment of hepatocellular carcinoma patients (Yau et al., 2013).

In addition to decreasing NO as a form of anti-cancer therapy, elevating NO to cytotoxic levels using NO donors has also been tried therapeutically. NO donors can potentially exert their anti-tumor activity by acting directly to reduce cancer progression, but also indirectly by increasing tumor blood flow to enhance the delivery of cytotoxic therapy to tumor tissue (Ning et al., 2014). Notably, the use of NO donors as a form of anti-cancer therapy has been challenging due to the short half-life of NO and the need to specifically target the right cells with the right dose. Thus, various NO donors are being tested in clinical trials as anti-cancer therapeutics, in combination with either chemotherapy, radiotherapy or immunotherapy, to overcome NO-related treatment difficulties as well as tumor cell resistance to conventional treatments (Huang et al., 2017b). Encouragingly, NO donors inhibit cultured human ovarian cancer cell survival and anti-apoptotic pathways, such as the NF-κB signaling cascade (known physiologically to be negatively regulated by S-nitrosylation), and sensitize drug-resistant tumor cells to apoptosis by both chemotherapy and immunotherapy (Bonavida et al., 2008; Garban and Bonavida, 2001; Marshall and Stamler, 2001). The NO donor glyceryl tri-nitrate has also been found to inhibit tumor progression in a Phase 2 study of prostate cancer patients following primary treatment failure (Siemens et al., 2009). Glyceryl tri-nitrate is also a promising chemo-sensitizing agent for advanced non-small-cell lung cancer (Dingemans et al., 2015), and for advanced rectal cancer, when used in combination with chemotherapy and radiotherapy (Illum et al., 2015). In another Phase 2 clinical trial, pretreatment of refractory lung cancer patients with the NO donor 1-bromoacetyl-3,3-dinitroazetidine (RRx-001) sensitized the patients to the chemotherapeutic drug carboplatin (Carter et al., 2016). Combining NO donors with other agents or anti-cancer drugs is another strategy that has proved to be effective against various cancer cell lines in preclinical models (Huang et al., 2017b). NO coupled to a non-steroidal anti-inflammatory molecule (NO-NSAID) induced apoptosis and modulated Wnt and NF-κB signaling in human colon cancer cells *in vitro* (Rigas and Kashfi, 2004), as well as *in vivo* in a breast cancer mouse model (Nath et al., 2015).

The mobilization of the immune system to treat cancer has taken a central stage in cancer therapy in recent years. A recent study demonstrated that hypoxia-induced expression of the immune inhibitory molecule programmed cell death ligand-1 (PD-L1) in murine melanoma cancer cells increased their resistance to lysis by *in-vivo*-generated cytotoxic T lymphocytes (CTLs), in a hypoxia-inducible factor-1α (HIF-1α)-dependent manner (Barsoum et al., 2014). Notably, as NO signaling activation was previously shown to prevent hypoxia-induced accumulation of HIF-1α (Barsoum et al., 2011), treatment with the NO donor glyceryl tri-nitrate prevented the hypoxia-induced expression of *PDL1* in murine melanoma cancer cells and diminished the cells' resistance to CTL-mediated lysis, indicating the potential use of NO donors as immunotherapeutic drugs against hypoxic tumor cells. However, treating hypoxic tumors with NO may be challenging, as NO was also demonstrated to enhance HIF-1α levels and activity in cultured human cancer cells (Berchner-Pfannschmidt et al., 2007; Kimura et al., 2000; Thomas et al., 2004).

## Conclusions

Since its discovery more than 200 years ago, numerous studies have identified NO as an important cellular signaling molecule involved in many physiological and pathological processes. Not surprisingly, NO is also emerging as a central player in cancer, due to its contribution to tumor initiation and progression. However, the pleiotropic nature of its physiological roles, its complicated spatial, temporal and dosage regulation at multiple levels, together with its short half-life, make NO challenging to target therapeutically. Indeed, a cell-specific approach is required to induce or inhibit NO synthesis for cancer therapy. Encouragingly, NO can also be regulated metabolically through the availability of arginine, making arginine a potential therapeutic target. Indeed, the activity of the key enzymes involved in NO production, namely ASS1, ASL, arginase and NOS, are frequently altered in various types of cancers, enabling us to identify vulnerabilities in NO-related pathways and to design novel anti-cancer drugs that target these enzymes. Recently, studies showed that the metabolic supplementation of citrulline, which drives NO synthesis (Kim et al., 2015), together with fisetin, which upregulates ASL and ASS1 levels, is a promising approach to overcoming NO-related tissue and dosage obstacles, and to restrict the development of inflammation-associated colon cancer (Stettner et al., 2018). In this approach, the body diverts the metabolic supplements to enable NO synthesis at the right place and at the required dosage. Undoubtedly, further advances in our understanding of the pathology of different cancers, and in the techniques for detecting NO, will strengthen our understanding of the ways in which arginine-NO metabolism contributes to cancer, and will aid in the development of related anti-cancer therapeutic approaches.

This article is part of a special subject collection ‘Cancer Metabolism: models, mechanisms and targets’, which was launched in a dedicated issue guest edited by Almut Schulze and Mariia Yuneva. See related articles in this collection at http://dmm. biologists.org/collection/cancermetabolism.
